# Investigating reduction of dimensionality during single-joint elbow movements: a case study on muscle synergies

**DOI:** 10.3389/fncom.2013.00011

**Published:** 2013-02-28

**Authors:** Enrico Chiovetto, Bastien Berret, Ioannis Delis, Stefano Panzeri, Thierry Pozzo

**Affiliations:** ^1^Section for Computational Sensomotorics, Department of Cognitive Neurology, Hertie Institute of Clinical Brain Research and Center for Integrative Neuroscience, University Clinic TübingenTübingen, Germany; ^2^Deparment of Robotics, Brain and Cognitive Sciences, Istituto Italiano di TecnologiaGenoa, Italy; ^3^UR CIAMS, EA 4532 - Motor Control and Perception Team, Université Paris-Sud 11Orsay, France; ^4^Department of Communication, Computer and System Sciences, University of GenoaGenoa, Italy; ^5^Institute of Neuroscience and Psychology, University of GlasgowGlasgow, UK; ^6^Center for Neuroscience and Cognitive Systems @ UniTn, Istituto Italiano di TecnologiaRovereto, Italy; ^7^Institut Universitaire de France, Université de Bourgogne, UFR STAPSDijon, France; ^8^INSERM U887, Motricité-PlasticitéDijon, France

**Keywords:** muscle synergies, non-negative matrix factorization, EMG, elbow rotations, dimensionality reduction, triphasic pattern

## Abstract

A long standing hypothesis in the neuroscience community is that the central nervous system (CNS) generates the muscle activities to accomplish movements by combining a relatively small number of stereotyped patterns of muscle activations, often referred to as “muscle synergies.” Different definitions of synergies have been given in the literature. The most well-known are those of synchronous, time-varying and temporal muscle synergies. Each one of them is based on a different mathematical model used to factor some EMG array recordings collected during the execution of variety of motor tasks into a well-determined spatial, temporal or spatio-temporal organization. This plurality of definitions and their separate application to complex tasks have so far complicated the comparison and interpretation of the results obtained across studies, and it has always remained unclear why and when one synergistic decomposition should be preferred to another one. By using well-understood motor tasks such as elbow flexions and extensions, we aimed in this study to clarify better what are the motor features characterized by each kind of decomposition and to assess whether, when and why one of them should be preferred to the others. We found that three temporal synergies, each one of them accounting for specific temporal phases of the movements could account for the majority of the data variation. Similar performances could be achieved by two synchronous synergies, encoding the agonist-antagonist nature of the two muscles considered, and by two time-varying muscle synergies, encoding each one a task-related feature of the elbow movements, specifically their direction. Our findings support the notion that each EMG decomposition provides a set of well-interpretable muscle synergies, identifying reduction of dimensionality in different aspects of the movements. Taken together, our findings suggest that all decompositions are not equivalent and may imply different neurophysiological substrates to be implemented.

## Introduction

A large amount of studies have provided in the last two decades evidence according to which the central nervous system (CNS) generates the muscle patterns necessary to achieve a desired motor behavior by combining a relatively small number of stereotyped spatial and/or temporal patterns of muscle activation, often referred to as “muscle synergies” (Bizzi et al., 2008). An appeal of this framework is that it suggests that the CNS may control movement execution through a relatively small number of degrees of freedom (dof).

Different conceptual definitions of muscle synergies have been given in the literature. These in practice translate into different mathematical models used to factor electromyographic (EMG) array recordings collected during the execution of variety of motor tasks into different kinds of temporal, spatial, or spatio-temporal organizations. Invariant temporal components (or “temporal synergies,” see Ivanenko et al., 2004, 2005; Chiovetto et al., 2010, 2012; Dominici et al., 2011) are defined as temporal muscle activation profiles that can be simply scaled and summed together to reconstruct the actual activity of each muscle. “Synchronous synergies” (Cheung et al., 2005, 2009, 2012; Ting and Macpherson, 2005; Torres-Oviedo and Ting, 2007, 2010) are stereotyped co-varying groups of muscle activations, with the EMG output specified by a temporal profile defining the timing of each synergy during the task execution. “Time-varying synergies” (d'Avella et al., 2003, 2006, 2008, 2011) are genuine spatiotemporal patterns of muscle activation, with the EMG output specified by the amplitude and time lag of the recruitment of each synergy.

Typically, previous studies about muscle synergies focused on a given decomposition that was then used to investigate potential functions of muscle synergies in complex motor tasks involving a large number of dof. Each of these decompositions has been used successfully to identify common physiologically important factors of muscle activity (Cheung et al., 2005; Ivanenko et al., 2005; d'Avella et al., 2006). The existence in the literature of multiple definitions of muscle synergies and their separate application to complex tasks complicates however the comparison and interpretation of the results obtained across studies, and it is not always clear why and when one synergistic decomposition should be preferred to another one. We propose instead here that the systematic study of the application of all these decompositions to the same and simple data set for which the mechanical action of each muscle contraction is well-known would greatly help to build intuition about the merit and functional interpretation of each synergistic decomposition. This would moreover be beneficial to the interpretation and comparison of different studies. We thus considered the extreme case of single-joint elbow movements, characterized by one kinematic dof, two antagonist muscles (biceps and triceps) and four experimental tasks (flexions and extensions along both the horizontal and vertical directions). We applied systematically decompositions into synchronous, time varying and temporal synergies of EMG data recorded during this elementary and well documented motor task (see Berardelli et al., 1996 for a review), whose biomechanical and neurophysiological bases were studied intensively (Gottlieb et al., 1995; Shapiro et al., 2005).

Our findings support the notion that each EMG decomposition provides a set of well-interpretable muscle synergies, identifying reduction of dimensionality in different aspects of the movements. Each temporal synergy indeed conveys information about a specific temporal phase of the movement (acceleration, deceleration, and stabilization). Synchronous and time-varying synergies instead encode respectively the simultaneous and coordinated actions of specific groups of muscles aiming to achieve a specific action goal and a task-related feature of the elbow movements (specifically the direction of motion). Taken together, our findings suggest that all decompositions are not equivalent and may imply different neurophysiological substrates to be implemented.

## Materials and methods

### Subjects

Eight healthy right-handed subjects (7 males, 1 female, ages 29 ± 4 years, mass 74 ± 9 kg, height 1.77 ± 0.07 m), participated voluntarily to the experiments that were all performed at the Robotics, Brain and Cognitive Sciences Department at Italian Institute of Technology (IIT) in Genoa (Italy). All subjects were in good health condition and had no previous history of neuromuscular disease. The experiment conformed to the declaration of Helsinki and informed consent was obtained from all the participants according to the protocol of the ethical committee of IIT.

### Protocol

**Figure 1:**
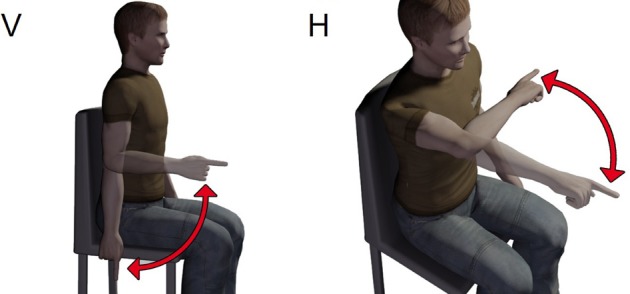
**Sketch of the experimental paradigm.** Subjects sat on a chair and had to accomplish flexions or extensions of the elbow along both the vertical (V) and horizontal (H) planes.

Subjects sat on a chair with their back straight and perpendicular to the ground. They were asked to perform one-shot 90° elbow rotations between two reference points along either a vertical and a horizontal plane (Figure 1). A total of four experimental conditions were thus studied (vertical flexion, VF; vertical extension, VE; horizontal flexion, HF; and horizontal extension, HE). For movements along the vertical direction, the two reference points were located in a vertical plane, placed laterally at approximately 10 cm from the subject's movement plane. To this aim, we used a wooden hollow frame containing 1.5 cm-spaced thin vertical fishing wires to which fishing leads indicating the requested fingertip initial position were attached. One reference point coincided with the subject's fingertip position in the vertical plane when the arm was completely relaxed and extended vertically with the index fingertip pointing at the ground (vertical position number 1, or VP1). The second point coincided with the subject's fingertip position in the vertical plane when, starting from VP1, the elbow was rotated of about 90° so that at the end the forearm was parallel to the ground (vertical position number 2, or VP2). The positions of the fishing leads were adjusted for each subject before the initiation of the experiment, based on the subject's upper arm and forearm lengths. For vertical elbow flexion subjects rotated the elbow so as to move their index finger from VP1 to VP2. On the contrary, during vertical elbow extension they had to move the fingertip from VP2 back to VP1. For rotation along the horizontal plane subjects sat in front of a table. One reference point on the table coincided with the horizontal location of the index fingertip when the upper-arm was kept horizontal with respect to the ground and perpendicular to the coronal plane and the forearm flexed of about 90° with respect to the upper-arm (horizontal position 1, or HP1). The second reference point coincided with the fingertip location when the whole arm was completely extended horizontally in front of the subjects and perpendicular to the coronal plane (horizontal position 2, or HP2). After that (for each subject) HP1 and HP2 were identified, their location was marked on the table by means of two small squared pieces of colored tape. The table plane laid 10 cm below the plane of rotation of the arm, avoiding thus to disturb the accomplishment of the movement. For horizontal elbow flexion subjects had to rotate the elbow so as to move their index finger from HP1 to HP2. On the contrary, during horizontal elbow extension they had to move the fingertip from HP2 back to HP1. Subjects were always asked to perform fast movements (mean velocities and average peak velocities are reported in Table 1 for each subject and condition). They performed 20 elbow flexion and 20 extensions for each plane orientation. During the experiment the wrist joint was frozen by means of two light and small sticks attached to the distal part of the forearm and the proximal part of the hand. At any trial repetition subjects put their index finger on the starting position. The experimenter started data acquisition and gave the “go” signal. The subjects performed the movement after the “go” signal and stopped on the target for about a second. Data acquisition stopped automatically after 2 s. At the end of the trial the subject assumed with his arm a relaxing position until the beginning of the next trial. After 20 trials subjects took a pause of about 3 min to avoid fatigue.

**Table 1:** **Average mean and peak angular velocities**.

	***HF***	***HE***	***VF***	***VE***
**PEAK VELOCITY (rad/s)**				
AL	8.86 ± 0.91	−8.95 ± 0.77	10.81 ± 1.12	−11.65 ± 2.37
AR	11.78 ± 0.97	−9.84 ± 1.29	12.51 ± 0.89	−12.55 ± 1.11
CA	9.69 ± 1.35	−8.26 ± 0.92	9.63 ± 1.66	−9.08 ± 2.59
MA	7.09 ± 0.99	−7.45 ± 1.83	7.18 ± 0.83	−7.92 ± 1.42
FR	4.85 ± 0.41	−7.37 ± 0.77	5.84 ± 1.07	−5.29 ± 0.57
FA	11.32 ± 1.01	−10.35 ± 1.03	9.72 ± 1.24	−12.04 ± 1.30
GI	7.96 ± 0.93	−8.17 ± 0.89	9.99 ± 0.69	−9.28 ± 1.48
LA	8.56 ± 2.10	−9.70 ± 0.68	10.19 ± 0.69	−12.63 ± 0.93
**MEAN VELOCITY (rad/s)**				
AL	3.26 ± 1.00	−3.47 ± 0.74	4.31 ± 0.68	−4.06 ± 0.98
AR	3.55 ± 0.34	−2.92 ± 0.83	3.95 ± 0.56	−3.69 ± 0.76
CA	3.86 ± 0.81	−3.65 ± 0.42	3.53 ± 0.60	−3.06 ± 0.64
MA	3.01 ± 0.37	−2.68 ± 0.49	3.00 ± 0.43	−2.81 ± 0.47
FR	2.04 ± 0.31	−1.61 ± 0.78	2.56 ± 0.35	−2.18 ± 0.47
FA	4.10 ± 0.92	−2.15 ± 0.54	3.68 ± 0.64	−3.54 ± 0.71
GI	3.41 ± 0.37	−3.65 ± 0.29	3.82 ± 0.63	−3.58 ± 0.54
LA	3.24 ± 1.89	−3.57 ± 0.60	3.66 ± 0.70	−3.47 ± 0.54

For each movement and subject the average velocities (± standard deviations) are reported. Averages and standard deviations were computed over all trial repetitions. The first entry of each row of the table indicates the initials of first and last name of a subject.

### Apparatus

During trials' execution kinematic data were recorded by means of a Vicon (Oxford, UK) motion capture system. Six passive markers were attached on subjects' right arm (the acromion process, lateral epicondyle of the humerus, the styloid process and the tip of the index finger) and head (external canthus of the eye and auditory meatus). EMG activity of biceps brachii (Bic) and triceps longus (Tri) was monitored by means of an Aurion (Milan, Italy) wireless EMG system. Impedance between the surface electrodes was always checked not to exceed 5 KΩ: in the case of higher values, skin was rubbed by means of an abrasive sponge in order to decrease it. EMG data were amplified (gain of 1000), band-pass filtered (10 Hz high-pass and 1 KHz low-pass) and digitized at 1000 Hz.

### Data pre-processing

Data were analyzed off-line using customized software written in Matlab (Mathworks, Natick, MA). Kinematic data were low-pass filtered (Butterworth filter, cut-off frequency of 20 Hz). The angular displacement of the elbow was computed starting from the markers' spatial positions. Elbow angular velocity of rotation was obtained by numerical differentiation of the angular position. Mean and peak angular velocities were computed for each trial. The mean velocity was computed as the mean value of the angular velocity over the movement duration. The time instants of movement initiation (*t*_0_) and end (*t*_*f*_) were defined respectively as the instants at which the bell-shaped angular velocity profile of the elbow exceeded and dropped below 5% of its peak value. For the EMG analysis, muscle signals were full-wave rectified, normalized in amplitude with respect to their maximum value recorded across all trials and conditions and low-pass filtered once more with a zero-lag Butterworth filter (cut-off frequency 5 Hz). The filtered EMG signals relative to each trial and comprised between 100 ms before *t*_0_ and *t*_*f*_ were normalized to a standard time window of 200 samples. By considering 100 ms before movement initiation we wanted to include in the analysis any kind of anticipatory activity associated with the movement. To identify specific invariant patterns characterizing the EMG activities of the different subjects, two versions of non-negative matrix factorization were applied to the low-pass filtered EMGs. The standard NMF algorithm (Lee and Seung, 1999) was used to identify both temporal components and synchronous synergies.

#### Temporal synergies (or temporal components)

NMF was applied to the matrix **M** of the EMG signals (size *m* by *T*, where *m* is the number of muscles signals and *T* the number of time samples), providing two matrices **U** and **C** (of dimension respectively *m* by *Nc* and *Nc* by *T*, where *Nc* is the number of temporal components) such that, at the time intant *t*, it results

(1) $$M(t)=\sum_{i=1}^{Nc}U_{i}{\text{C}}_{i}(t)+\text{residuals}$$

were **U**_*i*_ indicates the *i-th* column of the matrix **U** and C_*i*_(*t*) the *i-th* element of the column vector **C**(*t*). Note the number of muscles *m* indicates the number of muscles recorded during one single experimental trial. When considering multiple trials the matrix **M** was obtained by concatenating vertically the matrices of the single trials.

#### Synchronous synergies

NMF was applied to the transpose matrix **M**′ of **M**, providing thus two matrices **V** and **W** (this time of dimension respectively *T* by *Ns* and *Ns* by *m*, where *Ns* is the number of synchronous synergies) such that

(2) $$M^{\prime }(t)=\sum_{i=1}^{Ns}{\text{V}}_{i}(t)W_{i}+\text{residuals}$$

were V_*i*_(*t*) indicates the *i-th* element of the row vector **V**(*t*) and **W**_*i*_ the *i-th* row of the matrix **W**. Note that in (1) the *j-th* row of the matrix **M** results from the linear combination of the rows of the matrix **C** scaled by the scalar coefficients of the *j-th* row of the matrix **U**. Each row of **C** therefore contains one temporal component. In (2), conversely, the *j-th* row of **M**′ is obtained by combining linearly the rows of **W** scaled by the coefficients of the *j-th* row of **V**. Each row of **W** therefore, of dimension 1 by *Ns*, represents a vector of muscle activations, i.e., a synchronous synergy. Note also that, because of the constraints imposed by NMF on parameters, all the entries of the matrices **U**, **V**, **C**, and **W** are non-negative. Even in this case, when considering multiple trials before applying NMF the transposed of the matrixes of the single trials were concatenated vertically.

#### Time-varying synergies

We applied a customized version of standard NMF and that was developed by d'Avella et al. (2003) and d'Avella and Bizzi (2005). Similarly to standard NMF all the identified parameters are non-negative, but temporal shifts of the synergies are also allowed so that each column vector of **M** at the instant *t* is the following relationship is such that

(3) $$M(t)=\sum_{i=1}^{Nt}c_{i}w_{i}\left(t-\tau_{i}\right)+\text{residuals}$$

where *Nt* is the number of time-varying synergies and the *c*_*i*_ and τ_*i*_ are respectively the scaling coefficient and the time delay associated the synergy ***w***_*i*_. The algorithm by d'Avella et al. requires specifying the temporal duration of each time-varying synergy. In this study the time duration of each synergy was set, for each subject, as long as the time duration of the whole trial after time standardization (200 samples). Note that the residuals in (1), (2), and (3) decrease as the number of synergies increase. In case of multiple trials, the matrixes of the single trials were concatenated horizontally.

#### Selection of the number of synergies to be included in the EMG decomposition and their significance

In (1), (2), and (3) the numbers of muscle synergies (*Nc*, *Ns*, and *Nt*) are free parameters of the analysis that can be set arbitrarily by the experimenter. Here, it was decided to set in all the three cases the number of synergies according to a criterion based on the computation of the variance accounted for (VAF) as a function of *Nc*, *Ns*, and *Nt*. The VAF was defined as it follows

(4) $$\text{VAF=100}\cdot \left(1-\left({\left\|M\text{-}D\right\|}^{2}/{\left\|M-\text{mean}(M)\right\|}^{2}\right)\right)$$

where **D** is the matrix of the reconstructed EMG obtained by using a certain number of synergies and mean() is an operator that compute a matrix of the same size of the matrix **M** and whose rows are equal point by point to the mean values of the corresponding rows of **M**. The number of synergies was determined as the number of components at which the graph of the cumulative VAF presented a considerable change of slope (an “elbow”) and after which the slope of the graph became constant (Ferré, 1995). The exact point of change was quantitatively determined by using a linear regression procedure already used in literature (Cheung et al., 2005, 2009; d'Avella et al., 2006; Chiovetto et al., 2010, 2012). We computed a series of linear regressions, starting from a regression on the entire cumulative VAF curve and progressively removing the smallest value of number of component from the regression interval. We then compute the mean square residual error of the different regressions and selected the number of optimal synergies the first number whose corresponding error was smaller than 10^−3^. To minimize the probability to find local minima, we always ran NMF 25 different times on the same data set and consider as valid solution that provided the lowest reconstruction error between original and reconstructed error. To test the robustness and generality of the synergies extracted from each data set we exploited the two following cross-correlation procedures. We divided each data set in 5 parts of the same size. Since every data set consisted of the EMG activities of the Bic and Tri muscles collected during 20 repetitions of the same movement accomplished by one subject, each part consisted of the EMG activities of four trials. We then chose randomly 4 parts to use as training data set and one part as test data set. We extracted the synergies from the training data set and used them to reconstruct the activations of the test data set. We used the original and reconstructed test data sets to compute the VAF to draw the graph of the cumulative VAF. We also used the synergies extracted from each subject to reconstruct the EMG data sets of all the other subjects and assessed the level of reconstruction goodness by computing the VAF. For all cases, we verified that the extracted synergies did not result from a bias associated with the extraction methods by running a simulation. For each subject and decomposition, we compared the VAF values for the reconstruction of the experimental data obtained by combining the identified synergies with the VAF values of the reconstruction of random, structureless data reconstructed by combination of the synergies identified from those artificial data. Such data sets were generated by reshuffling the samples of each muscle independently in each trials of each subject. Reshuffled data were then low-pass filtered (5 Hz cutoff). For each one of the actual data set we simulated 50 artificial data sets and extracted the synergies by using the same procedure used for the observed data. We estimated the significance by computing the 95th percentile of the VAF distribution for simulated data.

#### Similarity of synergies across subjects

The similarity between synergies of different subjects was quantified by computing their scalar products. For synchronous synergies and temporal components we proceeded as follows. For all possible pairs of normalized synergies of two different subjects the corresponding scalar products were computed. Note that, by definition, such a product can only adopt values ranging between 0 and 1. The pair with highest similarity was selected and the corresponding synergies were removed from the two groups of synergies. The similarities between the remaining synergies were then computed, and the best matching pair of synergies was selected and removed from the original and reconstructed model. This procedure was iterated until all synergies were matched. To compute the similarity between time-varying synergies the procedure was very similar to the one just described above with the only difference that in the last case, before computing the scalar product, the matrices of the synergies were first rearranged by disposing the entries of the matrices in form of vectors. The similarity between synergies was then quantified by computing the maximum of the scalar products over all possible time delays of the second synergy with respect to the first. To access however the significance of the values of similarity provided by the scalar products we defined a similarity index (***S***) between two synergies. This index, ranging from 0 (similarity at chance level) to 1 (perfect matching of the synergies) was defined as follows

(5) $$S=\left(s_{\text{data}}-s_{\text{chance}}\right)/\left(1-s_{\text{chance}}\right)$$

where *s*_data_ is the scalar product between two synergies extracted from the actual data and *s*_chance_ is the mean scalar product between 200 pairs of random synergies. We generated the artificial synergies by resampling randomly from the distribution of the activation amplitude of each muscle in the data set from which the synergies were extracted and constructed sequences of random data with the same length of the extracted synergies. Artificial data were then low-pass filtered to match the smoothness of the actual data.

## Results

To compare systematically the results provided by different synergistic decompositions when characterizing the same EMG data set, we recorded EMGs during a series of elbow rotations and then we extracted and compared synchronous, time-varying and temporal muscle synergies.

**Figure 2:**
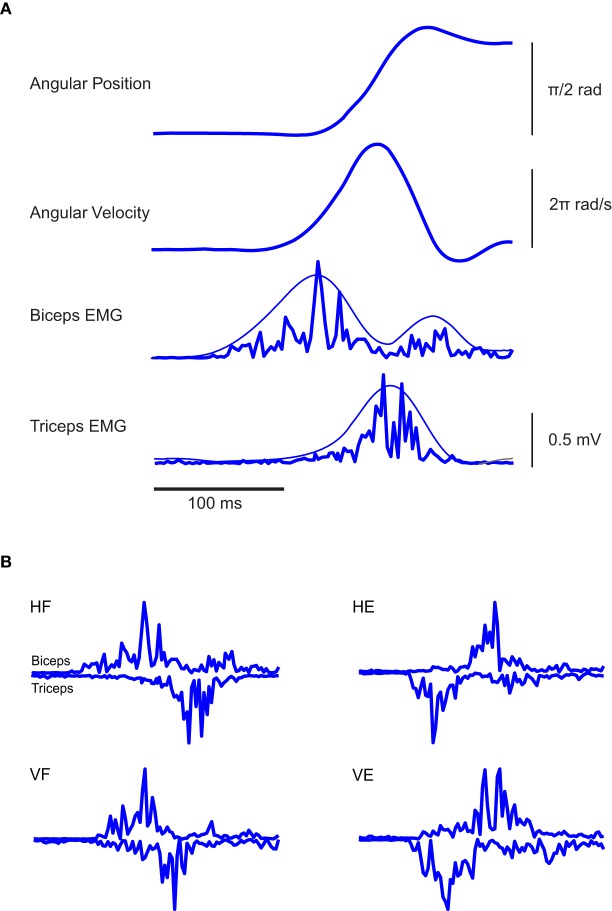
**Typical EMG and kinematic data associated with the experimental paradigm. (A)** From the top, angular elbow displacement and velocity associated with one typical elbow flexion in the horizontal plane are respectively depicted, along with the EMG activities of Bic and Tric muscles. In the two panels at the bottom, the smoothest lines represent the envelopes of the rectified EMGs and were obtained by low-pass filtering the rectified EMG at 5 Hz, the spikiest ones at 20 Hz. Clearly, different filtering frequencies do not modify the main temporal features of the signals. **(B)** EMG traces of individual rapid flexion and extension movements of the elbow in a normal subject. In all conditions the triphasic pattern results clearly present.

To illustrate the data, we begin by showing in Figure 2A the EMGs recorded during a typical trial accomplished by one subject and relative to an elbow flexion in the horizontal plane. Consistent with previous literature (Berardelli et al., 1996), such a movement is characterized by a sequence of three EMG bursts: an initial burst of the agonist muscle having the goal of providing the propulsive force to accelerate the movement, followed by a second burst of the antagonist to decelerate the movement and a third burst of the agonist to dampen the oscillation that other appears at the end of the movement. The latter final corrective action is also reflected in the final overshoot of the finger velocity profile. This sequence of bursts of activity was found also for elbow extension in the horizontal plane and flexion and extension in the vertical one (Figure 2B).

**Figure 3:**
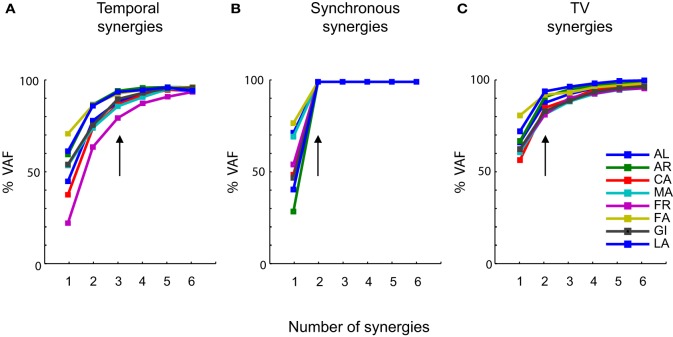
**Levels of approximation as a function of the number of synergies. (A)** Percentage of VAF as a function of the number of temporal synergies. **(B)** Percentage of VAF as a function of the number of synchronous synergies. **(C)** Percentage of VAF as a function of the number of time-varying synergies. Each colored line is associated to a specific subject (see most right panel), which in the figure is identified by the initials of his first and last name. In all the three panels the vertical arrows indicate the number of primitives at which the curves satisfy the linear regression criterion to choose the number of primitives (see “Materials and Methods”). These points are invariant across subjects and coincide, in most of the cases, with the points at which the curves present an “elbow” and start becoming straight.

We then considered the extraction of synergies from these data. The first interesting question is how many synergies of each type are needed to describe the data. The number of synergies to consider was determined, for each subject and type of decomposition, from the dependence of the percentage of VAF (see “Materials and Methods”) upon the number of synergies. The latter curves are plotted in Figure 3 for each type of synergy factorization and for each subject. The VAF curves in each decomposition were very similar across subjects. While for the temporal and time-varying decomposition we could extract up to 6 synergies (Figures 2A,C) we found that, when referring to a synchronous synergistic decomposition, two synergies were enough to account for 100% of the variance associated with the original data. We thus did not extract a number of synergies higher than two. In Figure 3B, however, we reported an amount of variance equal to 100% even for *N* = 3, 4, 5, and 6, to make Figure 3B graphically coherent with the other two panels, i.e., Figures 3A,B.

Figure 3A reports the VAF dependence upon the number of extracted temporal synergies. For all subject, the VAF reached a high value when including 3 synergies, and the linear interpolation algorithm that we used (see “Materials and Methods”) indicated that in all subjects 3 temporal synergies were sufficient to explain the vast majority of the variance (with additional temporal synergies generated by the NMF algorithm adding only a very small fraction of the total variance). The VAF curves for synchronous (Figure 3B) and time-varying (Figure 3C) synergies show that, for each individual subject, only two synergies were instead required to account for the variance of the EMG data.

**Figure 4:**
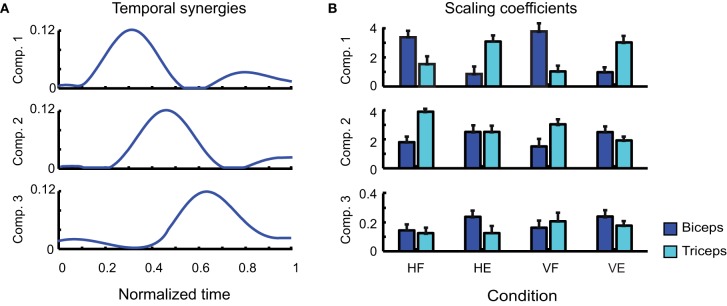
**Identified temporal synergies. (A)** Temporal components extracted from one typical subject (LA), ordered according to the time of the occurrence of their main peaks. **(B)** Corresponding scaling coefficients.

After having individuated their number, we next considered the shapes of the synergies extracted by each decomposition. Figure 4A reports the shapes of the three temporal synergies extracted from the EMGs of a typical subject (LA). The three temporal components clearly remind of the triphasic organization presented in Figure 2. Each temporal component is characterized by one major bump. The first temporal synergy can be interpreted as the component contributing the most to the modulation of the first burst of the agonist muscle during movement accomplishment: the second as the first burst of the antagonist; and the third as the second burst of the agonist. Note that the third temporal synergy shows an initial deactivation before the occurrence of the main peak. This initial part of the synergy can be associated to the antagonist deactivation, prior to movement initiation, of the anti-gravitational muscles during rotation along the vertical plane. The combination coefficients in Figure 4B (averaged across the repetitions of each kind of movement) show the contribution of each component to the activity of each muscle. Consistently with a triphasic pattern, it is evident that the first component is contributing more to the activity of the biceps during VF and HF; conversely, it contributes more to the activation of the triceps in VE and HE. Similarly the second temporal synergy is more active for the muscles opposing the actions exerted by the muscles activated by the first components. Thus for HF and VF movements the coefficients of the triceps are higher than those of the biceps. Whereas for VE the coefficient of the biceps is higher than that of the biceps, for HE movements however the level of the coefficients of the two antagonist muscles is approximately the same. The coefficients show then that, in all movements, the third component is contributing to the activations of both muscles in approximately equivalent proportion, in order to compensate for overshoots or to increase the joint stiffness by co-activating opposing muscles.

There are two points that need to be remarked. First of all in the pre-processing step all the EMG signals of each muscle were normalized with respect to the maximum value that was recorded for that muscle across all trials. Such a procedure may consequently lead to a partial loss of information about the relationship among the EMG amplitudes of different muscles monitored within the same trial. Moreover, trials were normalized in duration, which may introduce some supplementary temporal variability when merging all trials together to extract synergies. These can explain why the average coefficients of biceps and triceps relative to temporal synergy 2 in Figure 4B had approximately the same value for condition HE, differently from the expectation according to which the coefficient of the biceps should have appeared much larger than that of the triceps. According to the triphasic strategy, indeed, it should have been expected the second component to contribute mainly to the activation of biceps muscle which, in HE, is devoted to exert the antagonist role.

In addition, it is important to note that the number of identified temporal synergies is three, which is higher than the number of degrees-of-freedom to control (one joint angle, two muscles). This may look at first as a useless increase of complexity. However, the strength of a triphasic strategy in a single-joint motor task lies likely in its flexibility and power of generalization. Indeed, similar triphasic muscle organizations were found characterizing also arm raising (Friedli et al., 1984), rapid voluntary body sway (Hayashi, 1998) and whole-body reaching (Chiovetto et al., 2010, 2012) motor tasks. In accordance with this premise, one can note that the four tasks were all executed through a triphasic motor pattern. While previous studies mainly demonstrated the powerfulness of the synergy idea to reduce the dimensionality of motor control and execution, our results show in addition that temporal synergies present marked functional features.

**Figure 5:**
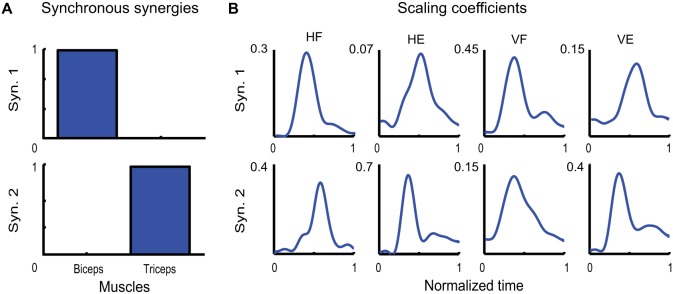
**Identified synchronous synergies. (A)** Synchronous synergies identified from one typical subject (LA). **(B)** Temporal evolution of the corresponding scaling coefficients.

Figure 5A depicts the two synchronous synergies extracted from the EMGs of a typical subject (LA). Each synergy is characterized by the activation of one single muscle. Due to their antagonist nature, biceps and triceps therefore were found to share no common level of activation. Note that, although such a result may seem trivial in a two dimensional space, we might have obtained a pair of linearly independent vectors characterized by noticeable activity of both muscles. In Figure 5B the temporal evolution of the scaling coefficients averaged across movement repetition are shown for each muscle and each movement. Note how, within each movement condition, the activities of the agonist and antagonist muscles are always characterized by one main burst in agreement with a classic triphasic pattern. Only for the first coefficient relative to HF movements the second burst is not clearly visible, this being very likely due to the averaging procedure.

**Figure 6:**
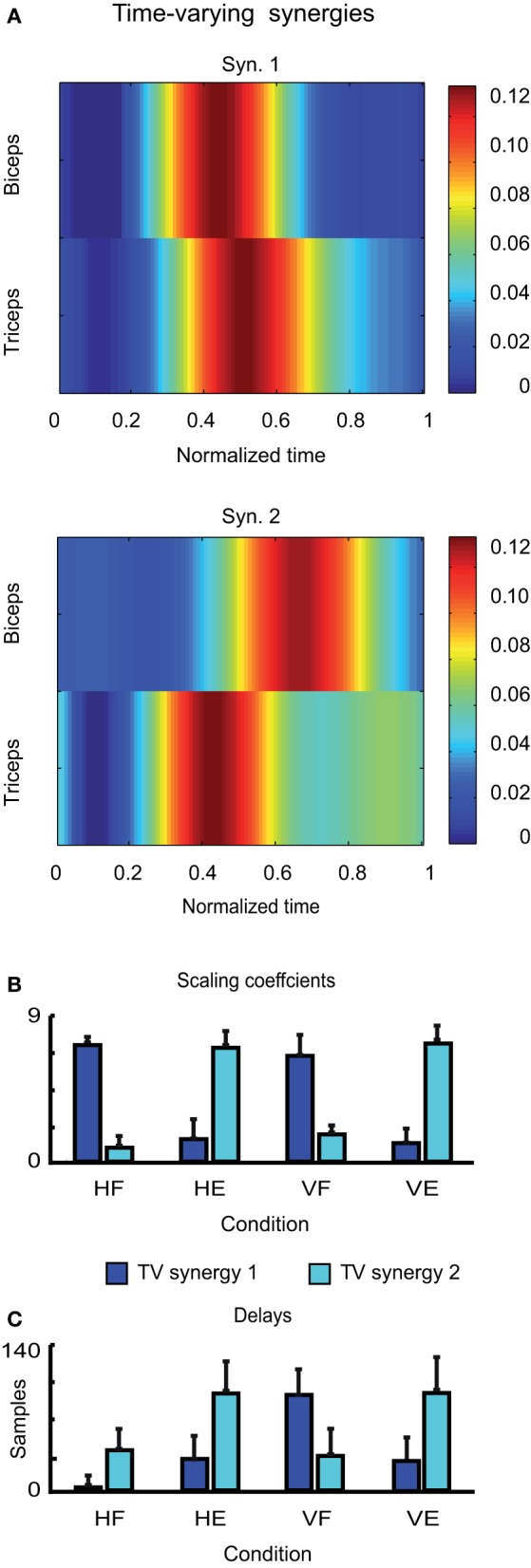
**Identified time-varying synergies. (A)** Time-varying synergies extracted from the EMG activity of one typical subject (LA). **(B)** Corresponding scaling coefficients. **(C)** Corresponding temporal delays.

Finally, the two time-varying synergies are shown in Figure 6A. They were characterized by one single burst for each muscle, one for the biceps and one of the triceps. The two synergies differed however for the temporal order in which the two burst occurred: whereas the burst of the biceps anticipated the burst of the triceps in the first time-varying synergy, the order of the peaks was reversed in the second one. The average scaling coefficients and temporal delays corresponding to each synergy are shown in Figures 6B,C. Note that also in this case, the contribution of each synergy to the EMG activity of each movement is consistent with the biomechanical feature of the movement itself. Thus time-varying synergy 1, in which the biceps is activated first, contributes more to HF and VF movements, while time-varying synergy 2, in which the triceps is activated first, contributes more to HE and VE movements.

In sum, we found that each kind of muscle decomposition provided a set of interpretable synergies. Each temporal component described a temporal phase of the movement. Each synchronous synergy described the simultaneous and coordinated action of a group of muscles (only one in our case) aiming to achieve a specific action goal. Each time-varying synergy related instead to a specific task-related variable (specifically a direction of motion).

**Figure 7:**
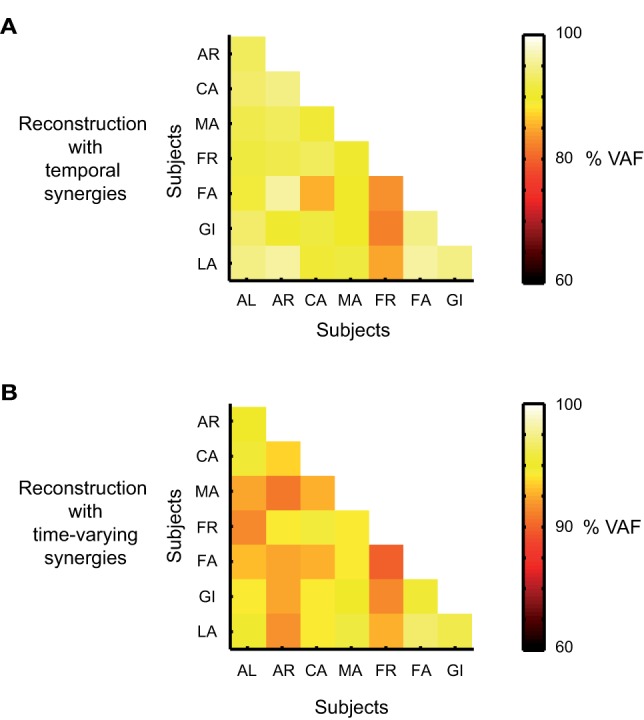
**Cross-validation results. (A)** Percentage of VAF for the reconstruction of the actual EMG data set of one subject by using the temporal synergies identified from the data sets of the other subjects. VAF values along each row are associated with the reconstruction of the data of one single subject. **(B)** Percentage of VAF for the reconstruction of the actual EMG data set of one subject by using the time-varying synergies identified from the data sets of the other subjects.

We used the synergies extracted from each subject to reconstruct the EMG data of each one of the others and assessed the percentage of VAF. The results are reported in forms of confusion matrices (Figure 7). The average percentage of VAF computed across subjects was 90 ± 7% when temporal synergies were extracted and used for reconstruction, and 87 ± 4 for the data sets reconstructed by using the time varying synergies. These values were found to be significant and did not result from a bias built in the extraction methods. The average 95th percentile of the distribution of VAF values obtained from the reconstructions of the simulated data were indeed much lower of the ones obtained from the reconstruction of the actual data, respectively 17.6 and 39.3% when data where decomposed according to the temporal and time-varying synergistic decompositions. The synchronous case was not considered given the features of the extracted sources and the fact that with such synergies a perfect match of the actual data could always be achieved.

**Figure 8:**
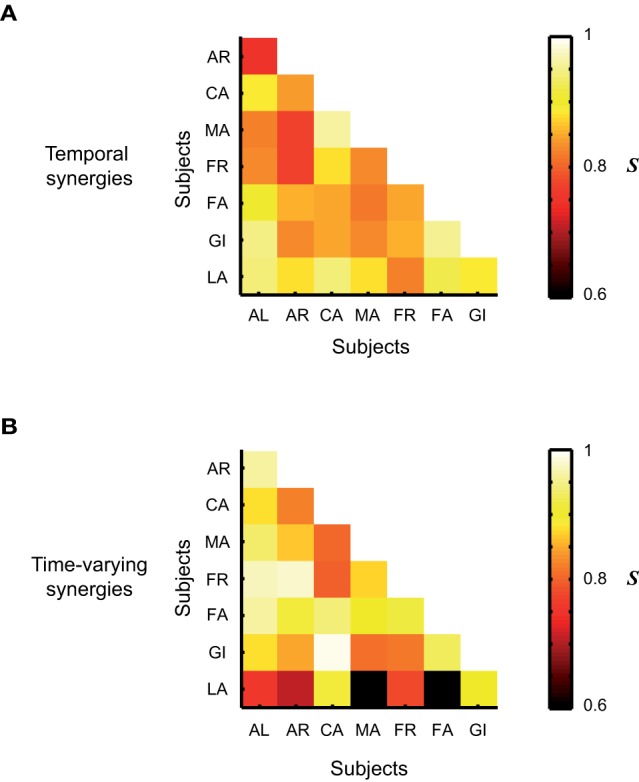
**Average level of similarity between groups of synergies identified from the EMG data of the 8 subjects that participated to the experiment. (A)** Similarity between groups of temporal synergies. **(B)** Similarity between groups of time-varying synergies. The average level of similarity between synchronous synergies is not shown as the identified set of synchronous synergies was the same across all subjects.

We quantified how much the synergies illustrated in Figures 4, 5, and 6 for one single subject were representative also of the synergies extracted from the EMG activity of the other subjects. To this purpose we computed the average scalar products and similarity indices between groups of synergies belonging to different participants. For the temporal components, the average scalar product was *s* = 0.93 ± 0.01, *s* = 1 ± 0 for the synchronous synergies and *s* = 0.91 ± 0.05 for the time-varying ones. The scalar products across subjects of synchronous synergies were always equal to 1 because for all the subjects the same set of synchronous synergies was always identified, in which only one single muscle was recruited at a time. Note that in this case also the similarity index ***S*** is always automatically equal to 1. The mean ***S*** values computed between groups of synergies extracted from different subjects are plotted in Figure 8. On average ***S*** = 0.86 ± 0.06 for the groups of temporal synergies and ***S*** = 0.85 ± 0.11 for the time-varying synergies. Note that in both cases the average similarity index was much higher than 0 (chance level). In sum, all synergies decompositions show a very high degree of robustness across subjects.

## Discussion

We used NMF-based methods to extract three different kinds of muscle synergies from the EMG activity of two antagonist muscles during the accomplishment of single-joint elbow rotations along both the horizontal and vertical planes. By using a well-understood motor task, we aimed to clarify better what are the motor features characterized by each kind of decomposition and to assess whether, when and why one of them should be preferred to another. We found well-defined interpretable results for each of the EMG signals decomposition considered. This allow us to discuss more in detail about what motor features each kind of muscle synergy decomposition encodes and, consequently, to explain why sometimes the extraction of one type of synergy may be more meaningful than another one.

In some previous studies (Ivanenko et al., 2005; Tresch et al., 2006) different unsupervised learning algorithms were applied to the same data set to verify the independence of the synergies from the particular technique used for their identification, or to test the superiority of an algorithm with respect to another one. In such studies however, all the algorithms used always relied on the same generative model, i.e., on the same definition of synergy. To our knowledge, this is the first study comparing synchronous, time-varying and temporal muscle synergies extracted from the same data set. Hence it offers the possibility to gain novel insights into the benefits provided by the different modular decompositions. Our choice of an elementary motor task for which most of the neuromuscular functions are well-understood, made the interpretations of various synergies as transparent as possible.

The results that we presented revealed that in all the cases NMF led to the identification of interpretable muscle synergies. The extraction of synchronous synergies yielded two primitives, each one characterized by the activation of only one of the two muscles, indicating that biceps and triceps (respectively flexor and extensor of the elbow joint) assumed independent levels of activation; in other words their activation waveforms did not, in general, co-vary in time. This might look like a trivial result given the small number of muscles considered and in view of antagonist nature of the two muscles during elbow rotations. However, following the generic definition of a muscle synergy as a group of muscles working together to achieve a common goal, it may appear surprising to find that the two main muscles controlling the task performance are not synergistic. However, the definition of synergies can be restated as groups of muscles acting at one or multiple joints to achieve a specific motor function (in our case the motor function could be simply flexing or extending the arm; in other terms, accelerate or decelerate the arm). From this point of view, our interpretation is in agreement with other previous studies considering more complex movements and a larger number of muscles. Similarly to us, for instance, the synergies extracted by Cheung et al. (2009) from the EMG activations of sixteen elbow and shoulder muscles of subjects performing a set of arm movements in space can be easily split in two groups: one encompassing synergies in which the most active muscles are flexor and another one in which extensor muscles are instead dominating (see Cheung et al., 2009, their Figure 3A). Also in this case, therefore, the goal associated with each synergy was to flex or extend the arm. By extension, this may suggest that muscles belonging to the same synchronous synergy share similarities with respect to their biomechanical function for the movement to be performed. Synchronous synergies were shown however encoding also other kinds of functional goals, or “strategies”. Torres-Oviedo and Ting (2007) extracted synchronous synergies from a set of leg and trunk muscles during a postural task and found synergies characterized mainly by activation of either ankle or knee muscle. These synergies resulted therefore in producing muscle activation patterns associated with two well-known postural strategies, usually referred to as “hip” and “ankle” strategies, which were previously deeply described in human postural control (Horak and Macpherson, 1996).

When extracting temporal muscle components the application of NMF provided a decomposition based on three temporal synergies. Each one of them was found playing a well-determined functional role during movement accomplishment, in agreement with the three movement phases present in the classical triphasic pattern (see Berardelli et al., 1996, for a review relative to elbow and wrist movements). The three phases can be resumed as follows: a first phase (coinciding with the first agonist EMG burst) to provide the impulsive force to initiate the movement, a second phase (antagonist burst) dedicated to halt the movement at the desired end-point and a third phase (coinciding with the second agonist burst) to dampen out the oscillations which might occur at the end of the movement. Although in a single-joint motor task such a triphasic strategy may look like a useless increase of complexity due to the fact that the number of synergies is higher than the number of muscles to control, its strength lies likely in its flexibility and power of generalization. Indeed, similar muscle organizations were found characterizing also arm raising (Friedli et al., 1984), rapid voluntary body sway (Hayashi, 1998) and whole-body reaching (Chiovetto et al., 2010, 2012) motor tasks. Along with the need of reducing movement complexity by reducing the number of dof (number of muscles), the decomposition of EMG activations based on the definition of temporal synergies showed that at some extent even the temporal dimension of the movement is a source of complexity that could be controlled and simplified by the CNS. These findings also pose the question of the neural implementation of this kind of temporal synergies. For single-joint rotations, Irlbacher et al. (2006) showed that the bursts composing the triphasic pattern were triggered in cascade with the possibility for the second burst to depend partly on what occurred during the first burst and not as a complete undividable sequence. This is compatible with the extraction of three temporal synergies to account for the control of elbow rotations across several conditions. However, this asks the question whether there are indeed three “spinal” temporal patterns recruited by different premotor drives or if the same temporal pattern is recruited by a delayed sequence of premotor drives. Interestingly, this idea of time shifts is present in the time-varying model of muscle synergies, which might have solved this issue.

We found that two time-varying muscle synergies could account quite well for the EMG activity associated with elbow movements. Each synergy was characterized by two main bursts of activation for both the biceps and triceps, whereas the time of occurrence of their peaks was inverted in the two synergies. While the burst of the biceps in the first synergy of Figure 6A occurs for first and may be thought to contribute therefore to start elbow flexion and the burst of the triceps to brake it, in the second synergies to role of the two muscles is inverted and the synergy is consistent with the pattern associated with an elbow extension. The two synergies seem therefore to intrinsically encode the direction of motion, or in other words, the motor task, and therefore may allow a hierarchical control of movements, in which task goals are only needed to be specified to generate complete muscle patterns. This finding is coherent with the results presented in previous investigations regarding arm movements (d'Avella et al., 2006, 2008, 2011) in which, even when a larger number of muscles was taken into account in the analysis, time-varying synergies where found to be directionally tuned, so that they resulted active only when the movements occurred in well-determined directions. We also stress the subtle difference between the interpretation of time-varying synergies and synchronous synergies: with the first time-varying synergy only flexions can be performed (maybe varying its speed or amplitude depending the way it is recruited). In contrast, the first synchronous synergy can be used for both flexion (to accelerate) and extensions (to decelerate), showing that both representations encode divergent aspects of the movements data set.

The use of very simple motor tasks characterized by well-known triphasic pattern allows us to evaluate some pros and cons of each of the decompositions used in this study. Previous works demonstrated that, in a triphasic pattern, the time of activation of the antagonist muscle is controlled independently by the cerebellum (Manto et al., 1995). Other studies (Cheron and Godaux, 1986) also reported that the timing of the antagonist burst onset increases with the movement amplitude, whereas the one of the agonist does not. Our results showed that neither the temporal synergistic decomposition nor the time-varying one can capture such timing features. In the first case, indeed, each one of the three bumps of Figure 4 is invariant in time and cannot be shifted temporally. This makes impossible to model the inter-trial variability of the onset of the antagonist muscle. Rather, each bump represents the average temporal evolution of the corresponding bursts across all trials. In the second case, differently, in each of the time-varying synergies that we identified from the experimental data set, the time lag between the activation of the two antagonist muscles is constant. This prevents the possibility, when reconstructing the data, to vary from trial to trial the time interval between the activations of the agonist and antagonist muscles, as observed in human subjects. Different considerations can instead be made for the results associated with the synchronous decomposition. As each synergy that was identified from the data is responsible for the recruitment of one single muscle indeed, the activation profile of each muscle can be set arbitrarily and independently for each trial. This allows therefore not only to model independently the times of activation of each burst in each trial, but also their amplitudes, in agreement with other experimental observations. Hannaford et al. (1985) demonstrated indeed that the first agonist burst is not modified by the vibration of the agonist muscle. In contrast the amplitude of the second agonist burst is increased and the vibration of the antagonist muscle increases the amplitude of the antagonist burst. Similarly to the synchronous one, even the temporal decomposition is suitable to capture such features of the amplitudes in the reconstructed data, as it allows the separate scaling of each one of the three identified bumps. The time-varying decomposition, on the contrary, introduces instead by construction a correlation between the amplitudes of the different muscles.

It was demonstrated that discrete movements regulated by a triphasic pattern may present an oscillatory component in the neural command (see for instance Cheron and Godaux, 1986). Very recently, it was also shown by the analysis of the dynamical structure of reaching movement that non-periodic movement such as the one presented here contains a strong rhythmic structure (Churchland et al., 2012). In this study the authors proved that, although EMG responses do not themselves exhibit state-space rotations, EMG can however be constructed from underlying rhythmic components. It makes thus sense to wonder which one of the decomposition methods that we investigated can be more useful or complementary for the understanding of the oscillatory nature of the control of movement. Each model might indeed provide a set of synergies revealing specific oscillatory features underlying the EMGs. In this framework, synchronous components cannot be of help, as they carry spatial and not temporal information. Interesting results might instead be provided by drawing the phase plots associated with each temporal component or with the activity of each muscle trace in a time-varying synergy. In case the plots presented evident rotations indeed, the hypothesis put forward by Cheron and Godaux and later by Churchland et al. would be strengthened. In the contrary case, however, the results obtained by these authors would not be discredited, as the absence of rhythmic features in the components might instead be due to the incapability of the synergy models to account for such features correctly.

We have in this discussion tried to provide evidence that the simple results that we found for the simple movement and system considered in this study might very likely hold also for more complex behaviors involving the action of large number of muscles. We think therefore that, in general, each kind of muscle synergy may encode a different motor feature. Specifically, temporal components encode different temporal phases of the movement, each one playing a specific functional role. Synchronous synergies encode the simultaneous and coordinated actions of specific groups of muscles aiming to achieve a specific motor function (e.g., accelerate the body toward the target). Finally, time-varying synergies encode high-level task-related functions (in this case the direction of motion). This suggests that the type of factorization to be chosen in each condition depends on which of these aspects the study intents to reveal. Note however that each type of synergies may not always characterize uniquely only one single motor feature, mainly because two or more variables may be correlated. Thus, for instance, the direction of motion can be inferred also from the amplitude of the scaling coefficients relative to temporal components (Figure 4B) once the action exerted by the muscles in known, or the triphasic temporal organization can be also reflected in the temporal evolution of the scaling coefficients in Figure 5B.

We conclude by stressing that a unifying synergy extraction method capturing all those aspects at once could simplify the interpretation of future works. If all these representations of synergies are simultaneously valid, then a more general model on the top of them should exist. Used systematically, such a model could allow better comparisons and interpretations of muscle synergy studies in more complex motor tasks.

### Conflict of interest statement

The authors declare that the research was conducted in the absence of any commercial or financial relationships that could be construed as a potential conflict of interest.
